# PSAURON: a tool for assessing protein annotation across a broad range of species

**DOI:** 10.1093/nargab/lqae189

**Published:** 2025-01-07

**Authors:** Markus J Sommer, Aleksey V Zimin, Steven L Salzberg

**Affiliations:** Department of Biomedical Engineering, Johns Hopkins University, Baltimore, MD 21218, USA; Center for Computational Biology, Johns Hopkins University, Baltimore, MD 21218, USA; Department of Biomedical Engineering, Johns Hopkins University, Baltimore, MD 21218, USA; Center for Computational Biology, Johns Hopkins University, Baltimore, MD 21218, USA; Department of Biomedical Engineering, Johns Hopkins University, Baltimore, MD 21218, USA; Center for Computational Biology, Johns Hopkins University, Baltimore, MD 21218, USA; Department of Computer Science, Johns Hopkins University, Baltimore, MD 21218, USA; Department of Biostatistics, Johns Hopkins University, Baltimore, MD 21218, USA

## Abstract

Evaluating the accuracy of protein-coding sequences in genome annotations is a challenging problem for which there is no broadly applicable solution. In this manuscript, we introduce PSAURON (Protein Sequence Assessment Using a Reference ORF Network), a novel software tool developed to help assess the quality of protein-coding gene annotations. Utilizing a machine learning model trained on a diverse dataset from over 1000 plant and animal genomes, PSAURON assigns a score to coding DNA or protein sequence that reflects the likelihood that the sequence is a genuine protein-coding region. PSAURON scores can be used for genome-wide protein annotation assessment as well as the rapid identification of potentially spurious annotated proteins. Validation against established benchmarks demonstrates PSAURON’s effectiveness and correlation with recognized measures of protein quality, highlighting its potential use as a widely applicable method to evaluate precision in gene annotation. PSAURON is open source and freely available at https://github.com/salzberg-lab/PSAURON.

## Introduction

Eukaryotic genomes continue to be sequenced, assembled and annotated at a rapid pace (1). Accurate annotation of these genomes remains a significant challenge, with errors potentially resulting in profound negative implications for downstream analyses. The quality of genome annotation, particularly of protein-coding gene annotation, is crucial for understanding gene function and for a wide range of applications in biotechnology and medicine (2).

Many metrics have been developed to assess the completeness and quality of genome assemblies. Merqury provides a *k*-mer-based assessment of quality, completeness and phasing for assemblies (3). Tools such as QUAST (4) and GAGE (5) provide metrics for assembly completeness and quality. BUSCO (Benchmarking Universal Single-Copy Orthologs) is a widely used tool that uses single-copy orthologs to estimate genome assembly and annotation completeness (6). OMArk uses orthologous protein information to quantify genome completeness, assess taxonomic consistency and detect contamination from taxonomically inconsistent proteins (7). No alignment-free, *k*-mer-free, reference-free method currently exists to assess the precision of annotation.

Here we introduce PSAURON [Protein Sequence Assessment Using a Reference ORF Network; pronounced ‘Sauron’ (8)], a novel software tool that accurately scores predicted protein sequences and enables the genome-wide evaluation of protein-coding gene annotation. Using a temporal convolutional network (TCN), a machine learning model that we previously applied to prokaryotic genome annotation (9), PSAURON analyzes coding sequences (CDSs) to assess the likelihood that a given annotation correctly identifies protein-coding genes. We trained the PSAURON TCN on a comprehensive dataset of protein sequences from a broad range of plant and animal genomes, enabling it to recognize general patterns indicative of protein-coding sequences without the need for retraining on any individual species. One benefit of PSAURON lies in its ability to quickly assess whole-genome annotations with low computational requirements, providing researchers with a score that reflects the confidence in annotation accuracy for each protein-coding sequence. This scoring system is designed to highlight potential inaccuracies in annotations, facilitating the identification of sequences that may require further investigation.

We validated PSAURON against well-established reference datasets and protein quality metrics to demonstrate its effectiveness in distinguishing between high- and low-quality gene annotations. As more genomes continue to be sequenced, the need for accurate and efficient tools for assessment of proteins will become increasingly critical. PSAURON represents a step forward in this regard, offering to the genomics community an efficient solution for quality control in protein-coding gene annotation.

## Materials and methods

### Model architecture

**Figure 1.**
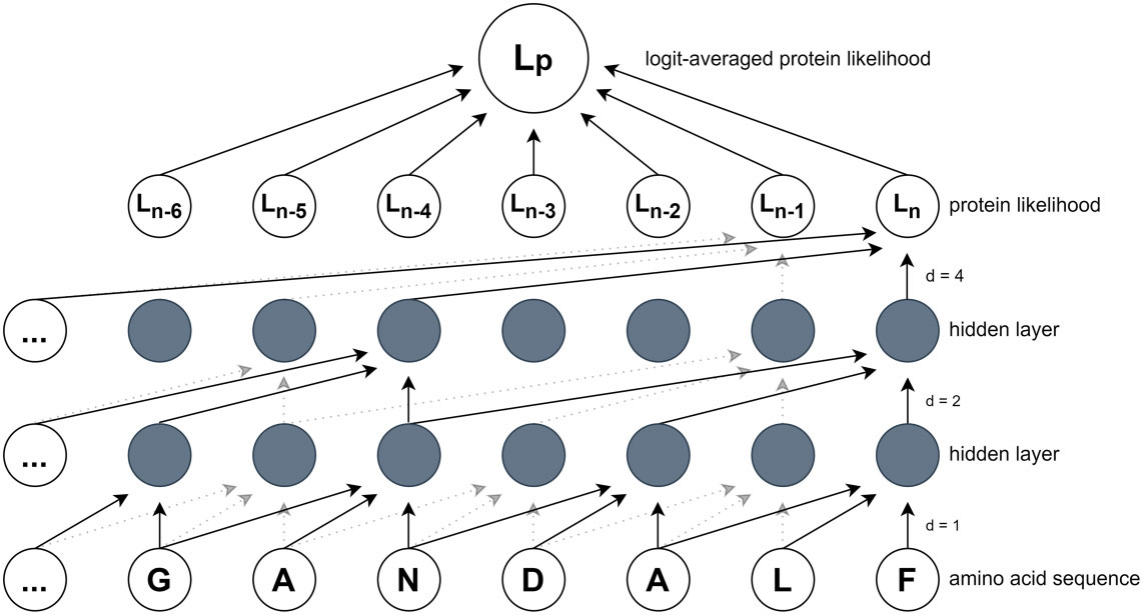
Model architecture. The pretrained PSAURON TCN model produces a sequence of likelihoods for any length amino acid sequence. These likelihoods are logit averaged into a single protein likelihood, *L*_p_, shown at the top. The simplified model shown here for demonstration purposes has two hidden layers, a kernel size of 3, and dilation factors of 1, 2 and 4. Solid lines represent the path of information used to calculate *L*_n_, while dotted lines are used to calculate *L*_*n−*1_. Left zero padding ensures that all layers are of equal size. The actual PSAURON TCN model uses six layers, 32 hidden units per layer, a kernel size of 16 and a dilation expansion factor of 2, for a total of 383 457 trainable parameters.

The TCN architecture used here is based on one developed by Bai *et al.* (10) and modified to work with protein sequence data in Balrog, a bacterial gene finder (9). Figure 1 highlights some unique aspects of the TCN that make it amenable to working with amino acid sequence data, particularly its ability to produce a single predicted likelihood score for any length protein.

The PSAURON TCN belongs to a family of convolutional network architectures (10) that use dilated convolutions and left zero padding to capture information from a wide receptive field, mapping input sequence data to an output of the same length. For our data type, amino acid sequences, the TCN architecture enables us to infer predicted likelihood for any length protein. No calibration was performed, so outputs represent predicted likelihoods that a sequence is in-frame protein, given that the sequence is protein-coding. The PSAURON TCN (Figure 1) uses six layers, 32 hidden units per layer, a kernel size of 16 and a dilation expansion factor of 2, for a total of 383 457 trainable parameters. These hyperparameters were chosen to be similar to those used in Balrog, where Bayesian hyperparameter optimization was performed (9,11). To compare scores between proteins of varying lengths, we use the logit-averaged likelihood, a single number between 0 and 1 that represents an estimate of the predicted protein’s likelihood. Whereas the arithmetic average treats all likelihoods close to 0 or 1 as nearly identical, logit averaging more heavily weights likelihoods that are close to 0 or 1. For example, the arithmetic average of 0.500 and 0.999 is ∼0.75, whereas the logit average is ∼0.97. Thus, if any part of a sequence appears protein-like while the model is unsure of the rest of the sequence, then PSAURON will be more likely to assign a high score. The logit average for a sequence ${\boldsymbol{p}}$ of length ${\boldsymbol{n}}$ is defined as


(1) \begin{equation*}{\boldsymbol{{\rm logit}\ {\rm average}}} = \ \frac{1}{{1 + {{{\rm e}}^{ - \frac{1}{n}\mathop \sum \nolimits_{i = 1}^n \ln \left( {\frac{{{{p}_i}}}{{1 - {{p}_i}}}} \right)}}}}\end{equation*}

### Model training

The PSAURON TCN model was trained on CDSs from all plant and animal genomes available from the NCBI RefSeq database as of August 2023. These 1090 eukaryotic genomes included 171 plants, 359 invertebrates, 214 mammalian vertebrates and 346 other vertebrates. Accession numbers for all genomes can be found in Supplementary Table S1. Sequences were excluded from analysis if they contained annotated in-frame stop codons, started with a non-standard start codon, contained non-ACGT nucleotides or had a length that was not divisible by 3. This selection process generated 33 869 774 unique CDSs.

Subsequences of 100 amino acids (aa) overlapping by 50 aa (shingles) were extracted from protein sequences translated from annotated-frame and out-of-frame CDSs to generate positive and negative data, respectively. For all shingles, start and stop codons were removed to prevent the learning algorithm from learning to label a sequence based on the presence or absence of those codons. No stop codons were kept in any data used to train or test PSAURON. Shingles were generated from 1 000 000 CDSs (2.95%) selected at random from the full CDS dataset. From this subset, annotated-frame shingles numbered 11 406 219. The same number of out-of-frame shingles were randomly selected to maintain equal class balance during model training. The model was trained with binary cross-entropy loss with annotated-frame CDSs as positive data and out-of-frame CDSs as negative data, with a batch size of 1000. Adaptive moment estimation with decoupled weight decay regularization (AdamW) (12) was used to minimize loss during training. Model training required over 8 h of computing time on a Google Colab server with an NVIDIA V100 16GB GPU.

### Protein sequence assessment

PSAURON assesses proteins by calculating a score between 0 and 1 for any given amino acid sequence, where 1 indicates that the sequence is a *bona fide* protein. The model is trained to maximize the difference in scores between sequences translated from in-frame CDS, which should receive scores close to 1, versus out-of-frame CDS, which should receive scores close to 0. Amino acid sequences that bear no resemblance to either in-frame or out-of-frame protein translations may receive unpredictable scores.

By default, PSAURON uses scores from all six frames of the CDS to determine whether a sequence encodes a protein, and it intentionally ignores stop codons. Thus, a high PSAURON score does not guarantee that a sequence contains a valid open reading frame (ORF). Rather, because the model was trained to recognize both in-frame and out-of-frame translated sequences, scores from all frames are used to maximize predictive performance. For a sequence to be assessed as ‘positive’, the in-frame score must be >0.5 and the mean out-of-frame score must be <0.5. These parameters may be changed by the user, and scores for all frames of all sequences are provided in the default output file. PSAURON can also run directly on protein sequence with no CDS provided, and in this case will provide only the score of the annotated frame.

### Proteome-wide annotation assessment

In addition to scoring each protein individually, PSAURON also provides an overall score between 0 and 100 for the entire input annotation. This score represents the percentage of proteins provided in the annotation that receive a ‘positive’ assessment as described above. The whole-proteome PSAURON score can change depending on user-defined parameters that trade sensitivity for precision. The whole-proteome score and scores for each annotated protein sequence are provided in the default output file, and these may be used to sort proteins by PSAURON’s confidence.

## Results

### Reference proteome analysis

Table 1 provides whole-proteome PSAURON scores for a variety of model organisms, including animals, plants, fungi and prokaryotes. Supplementary Tables S2 and S3 contain PSAURON scores for all proteins in the Matched Annotation from NCBI and EMBL-EBI (MANE) annotation as well as the UniProt reference rice proteome. For these evaluations, PSAURON was run in single-frame or protein mode with a score cutoff of 0.5 and no minimum protein length threshold.

**Table 1.** Reference scores: proteome-wide PSAURON scores for selected UniProt reference proteomes and human genome annotation databases

Genome	# Proteins	Proteome-wide PSAURON score
Animal		
* H. sapiens*, RefSeq^a^	136 194	97.7
* H. sapiens*, GENCODE^a^	111 048	92.7
* H. sapiens*, CHESS^a^	106 188	96.8
* H. sapiens*, MANE	19 352	97.6
* C. elegans*	19 827	96.4
* R. norvegicus*	22 897	95.0
* M. musculus*	21 702	96.6
* D. melanogaster*	13 824	97.5
Plant		
* A. thaliana*	27 448	95.3
* G. max*	55 855	91.9
*** O. sativa***	**43 667**	**73.8**
Protist		
* P. falciparum*	5361	94.3
* D. discoideum*	12 718	95.7
Fungus		
* C. albicans*	6035	98.1
Archaeon		
* M*. *jannaschii*	1787	99.4
Bacterium		
* E. coli*	4403	97.2

^a^Annotations contain more than one isoform per gene locus; all other annotations contain one protein per gene locus.

*H. sapiens:* RefSeq GCF_000001405.40, GENCODE 45, CHESS 3.01, MANE 1.3; *C. elegans*: UP000001940; *R. norvegicus*: UP000002494; *M. musculus*: UP000000589; *D. melanogaster*: UP000000803; *A. thaliana*: UP000006548; *G. max*: UP000008827; *O. sativa*: UP000059680; *P. falciparum*: UP000001450; *D. discoideum*: UP000002195; *C. albicans*: UP000000559; *M. jannaschii*: UP000000805; *E. coli*: UP000000625. PSAURON was run in protein mode with the default score threshold of 0.5; the proteome-wide PSAURON score in this table represents the percentage of proteins that scored above 0.5. All proteome-wide PSAURON scores are >90 with the sole exception of rice, *O. sativa*, in bold.

**Figure 2.**
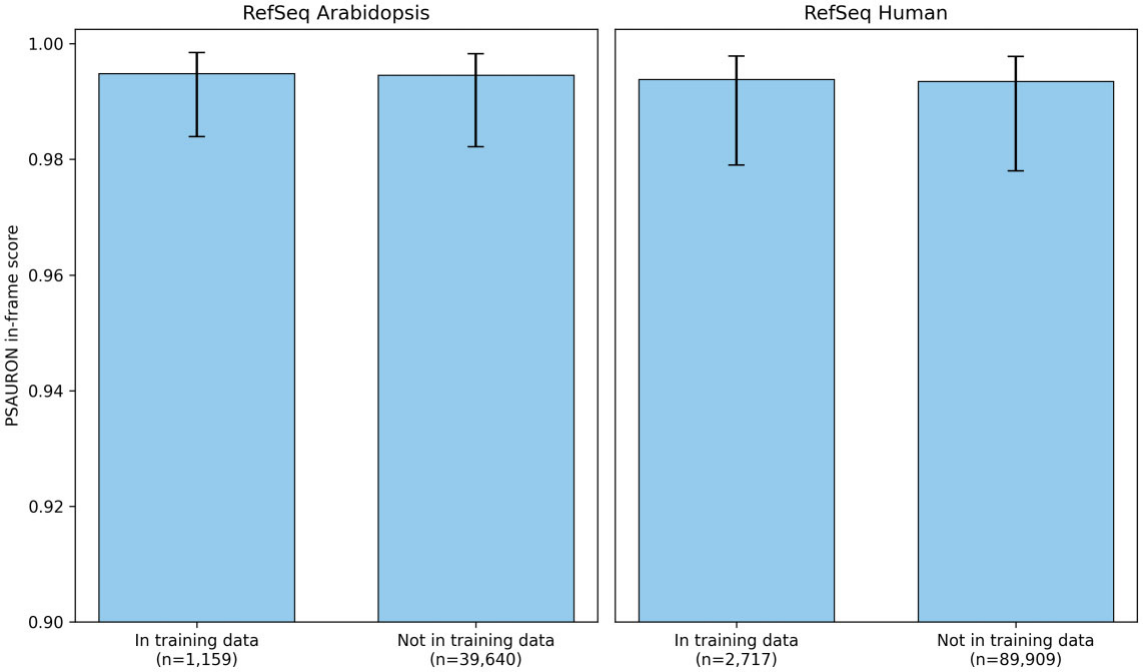
Training versus non-training CDS scores. Distributions of PSAURON scores, shown by the median and IQR, are shown for *Arabidopsis* and human CDSs included and excluded from the training data. The median (25th percentile, 75th percentile) values for each group are as follows: *Arabidopsis* training, 0.994 (0.977, 0.998); *Arabidopsis* non-training, 0.992 (0.972, 0.998); human training, 0.994 (0.978, 0.998); human non-training, 0.993 (0.972, 0.998). No statistically significant difference in scores between training and non-training data was found.

PSAURON was trained on all plant and animal genomes available from the NCBI RefSeq database as of August 2023. No eukaryote was explicitly excluded from the training dataset, including those shown in Table 1. However, only 1 000 000 (2.95%) of the 33 869 774 total RefSeq CDSs were randomly selected to be used during training. Figure 2 shows the median and interquartile range (IQR) distribution of PSAURON scores for all RefSeq human and *Arabidopsis* CDSs, separated by those included in the training data and those not included in the training data. Mann–Whitney *U* tests were used to compare scores of training versus non-training data in *Arabidopsis* and human. No statistically significant difference in scores was found: human, *P* = 0.12; *Arabidopsis*, *P* = 0.22.

Interestingly, despite PSAURON having been trained exclusively on eukaryotic proteins, the highest score in Table 1, 99.4%, was achieved by a low-GC-content archaeon, *M. jannaschii*. Prokaryotic annotation in low-GC genomes is highly accurate, and PSAURON agrees with the annotation for nearly all the proteins in this genome. All other reference proteomes achieved scores >90%, with the sole exception of *Oryza sativa* subsp. *japonica* (rice), which scored 73.8%. This whole-proteome score suggests that nearly 30% of the annotated proteins in rice might be incorrect.

To further explore the source of the low-scoring proteins in rice, we compared the EnsemblPlants *O. sativa* annotation and RefSeq *O. sativa* annotation. Ensembl annotates 42 582 proteins, of which 31 982 (75.1%) are complete, i.e. have both start and stop codons. RefSeq annotates 42 936 proteins, of which 42 727 (99.5%) are complete. Due to the lack of valid start and/or stop codons, incomplete proteins in the Ensembl annotation are more likely to be in the wrong reading frame. The RefSeq annotation used Gnomon (13), the NCBI eukaryotic gene prediction pipeline, while the EnsemblPlants gene models were imported from the Rice Annotation Project (RAP-DB) (14,15). RAP-DB used a combination of full-length complementary DNA from rice; transcript data from 149 monocot species, including wheat, barley and corn; 6 700 357 expressed sequence tags from the DNA Data Bank of Japan; 1 433 269 protein sequences from UniProt and RefSeq plant species; and predictions from multiple *ab initio* gene finders.

Of the 10 600 incomplete proteins in the Ensembl rice annotation, 3513 have no blastp alignment to any protein in the RefSeq rice annotation. A manual check of a few of these proteins against the non-redundant protein database showed only self-matches, with no hits to any other species’ proteins. Based on the lack of start and stop codons, alongside the alignment evidence, it seems many of the low-PSAURON-scoring rice proteins are likely annotated in the wrong reading frame.

### Comparison of PSAURON scores and protein structure predictions

The AlphaFold2 program has been demonstrated to be remarkably accurate at predicting the three-dimensional structure of many proteins (16,17). AlphaFold2 also produces a score for each structure, known as the predicted local distance difference test (pLDDT), which indicates the program’s confidence in its structural prediction. High-scoring structures are likely to be correct, while lower-scoring structures, with scores below 70, are ‘considered low confidence and should be treated with caution’ (17). Not all proteins fold into a stable structure or receive a high pLDDT from AlphaFold2; for example, for the human genome ∼58% of proteins fall into the high-confidence group, with scores above 70 (18). Despite these caveats, the accuracy of AlphaFold2 makes it a powerful tool for independently evaluating the quality of a set of predicted proteins, as we have demonstrated previously (19).

**Figure 3.**
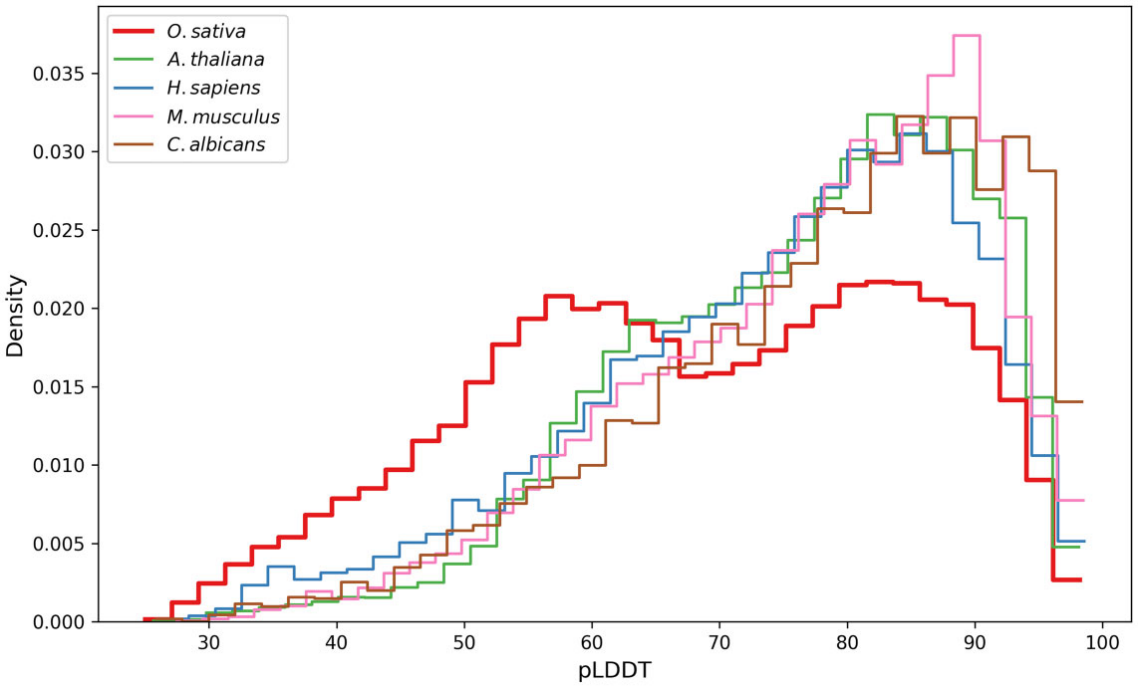
AlphaFold2 confidence distributions of reference proteomes. Histograms of AlphaFold2 pLDDT score of proteins in UniProt reference proteomes. High-quality eukaryotic proteomes tend to show similar distributions of scores. The rice proteome is an outlier with a bimodal pLDDT distribution.

For the experiments here, we hypothesized that the scores assigned by AlphaFold2 should follow a similar distribution across a wide variety of organisms. Figure 3 shows the distributions of pLDDT scores for five eukaryote genomes, including two animals (human and mouse), two plants (rice and the model plant *Arabidopsis thaliana*) and one fungus. As expected, all but one of the distributions look similar. The sole outlier in this analysis is the rice proteome, shown in red, which has a secondary peak in pLDDT density near 60, while all other proteomes peak near 90.

Worth noting is that we obtained this low proteome-wide PSAURON score for rice on the UniProt reference proteome annotation, which is based on the Rice Annotation Project Database (14,15), but not for the RefSeq rice annotation, which we scored at 96.0. Both annotations use the same rice genome assembly, which suggests that the issue with the rice proteome is a result of flawed annotation rather than assembly.

**Figure 4.**
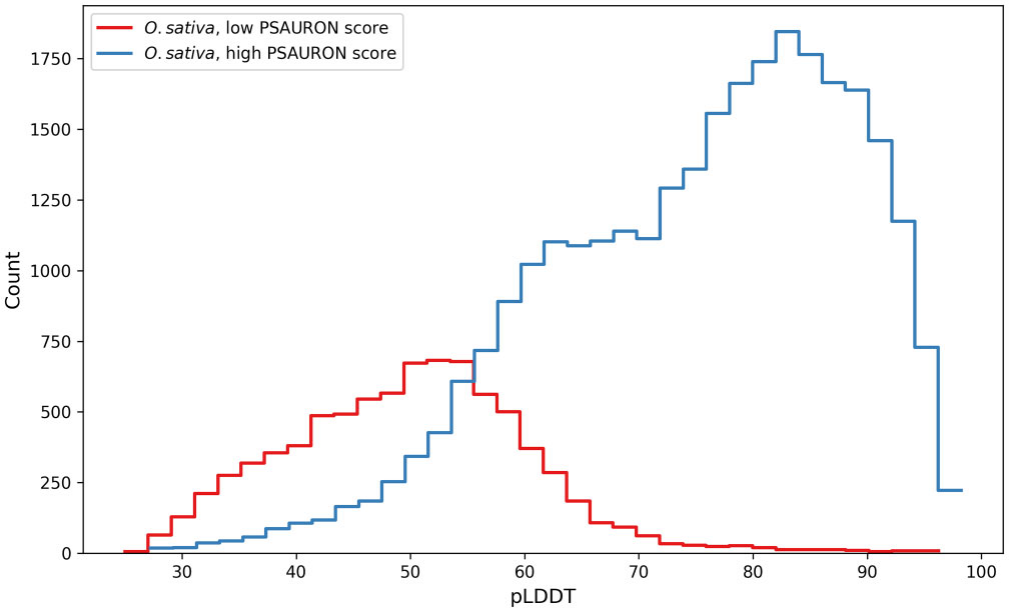
Rice reference proteome AlphaFold2 predictability separated by PSAURON score. Histograms of AlphaFold2 pLDDT scores of the rice reference proteome. The blue (right) curve shows proteins with high PSAURON scores (>0.8) and the red (left) curve shows proteins with low PSAURON scores (<0.2). 96.9% of all low-PSAURON-scoring proteins receive a pLDDT below 70. The pLDDT distribution of high-PSAURON-scoring proteins in the rice proteome more closely resembles those of other high-quality reference proteomes.

As shown in Figure 4, rice proteins with low PSAURON scores also tended to receive low AlphaFold2 confidence scores, with 96.9% of PSAURON’s low-scoring proteins receiving a pLDDT below 70. Note that PSAURON was trained on CDS sequences alone and was given no information on protein structure. Thus, in combination with unusually low structure scores, the current UniProt rice proteome annotation appears to contain several thousand proteins that may be misannotated. Interestingly, if one were retain only the proteins to which PSAURON assigned a high score, then the distribution of AlphaFold2 scores for rice (blue curve in Figure 4) would more closely resemble the other distributions shown in Figure 3.

### Consistency with manual curation (*Arabidopsis*)

The *Arabidopsis* Information Resource (TAIR) provides a curated ranking system for genes in the *A. thaliana* reference genome, ranking them from zero to five stars based on transcriptomic and proteomic data as well as multiple sequence alignment and genomic conservation analysis (20). Using the TAIR10 data, we scored all proteins with PSAURON in single-frame mode to generate PSAURON score distributions for each ranking group. Using a consumer-grade Dell XPS 13 laptop running Ubuntu 20.04 LTS with a 4-core Intel i7-1185G7 at 3.00 GHz, PSAURON scored the full TAIR10 set of 35 386 proteins in 132 s with a peak memory usage of 1.95 GB.

**Figure 5.**
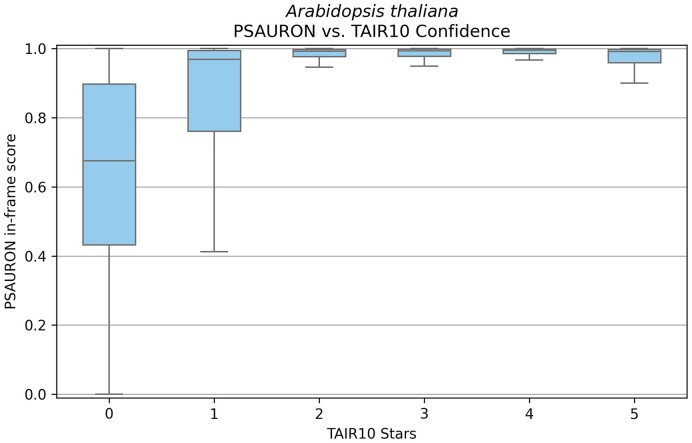
PSAURON scores for *Arabidopsis* proteins by TAIR10 confidence. Box and whisker diagram of the PSAURON score distribution for annotated CDSs in the TAIR10 reference genome. The *x*-axis shows the TAIR confidence ranking for each group of genes, ranging from a minimum of zero (low confidence) to a maximum of five stars. The boxes show the IQR of individual protein PSAURON scores. The whiskers extend to the furthest point within 1.5 times the IQR.

The PSAURON scores were consistent with the TAIR confidence rankings: as shown in Figure 5, the higher-confidence proteins (2–5 stars) received very high PSAURON scores with a tight distribution of values close to 1.0. For low-confidence proteins with one- or zero-star confidence ratings, PSAURON produced lower average scores with a wider distribution, particularly for the zero-star proteins. Thus, the independently determined manual curation of *Arabidopsis* supports the scores assigned by PSAURON.

### Analysis of long non-coding RNA

To assess the performance of PSAURON on true negative sequences, i.e. sequences that contain no valid protein, we used human long non-coding RNA (lncRNA) transcripts from LNCipedia version 5.2 (21). We analyzed only high-confidence transcripts with putative protein coding genes excluded, resulting in 107 039 transcripts from 49 372 genes. For the purposes of this analysis, any protein found in the high-confidence set of lncRNA transcripts is referred to as a false positive.

**Figure 6.**
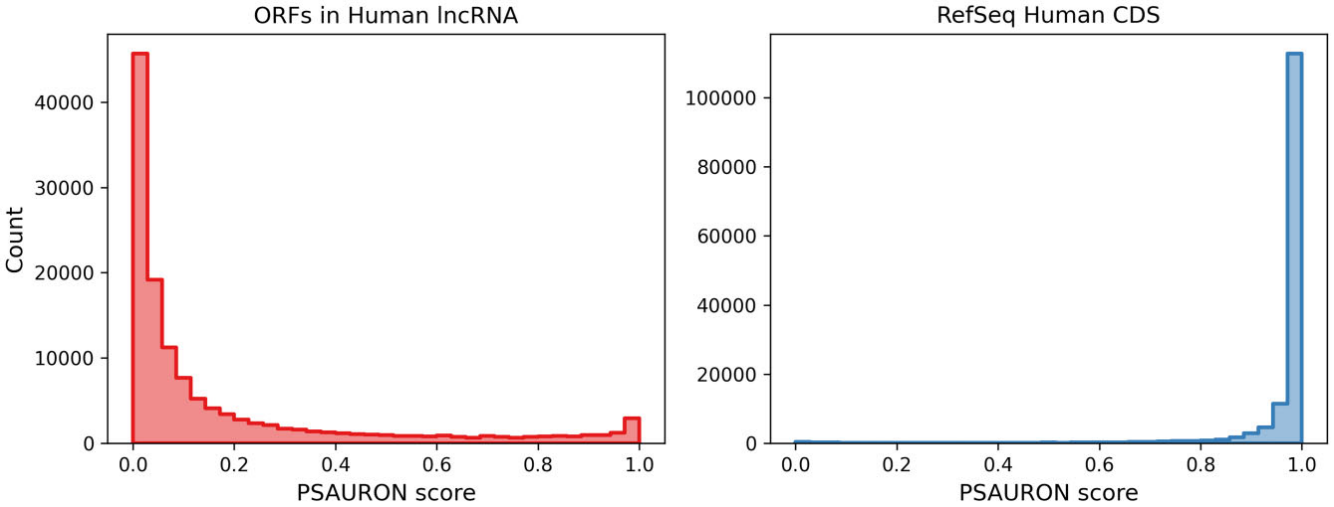
PSAURON scores of lncRNA versus CDS. ORFs in lncRNA receive lower PSAURON scores than CDSs. Thirteen percent of ORFs in lncRNA receive a score >0.5, while 2.3% of RefSeq CDSs receive a score below 0.5. Not all ORFs with a score >0.5 are classified as proteins, as PSAURON also uses the five out-of-frame scores when classifying sequences.

Before running PSAURON, ORFs must be found within transcript sequences. Parameters of ORF selection were matched as closely as possible to GeneMarkS-T (22). All ORFs with a valid stop and a standard or alternative start codon, in any forward or reverse reading frame, were used. Additionally, ORFs were allowed to run off either or both ends of the transcript as 5′ partial, 3′ partial, or internal ORFs. To ensure consistency with GeneMarkS-T, the minimum ORF length was set to 294 nucleotides not including the stop codon, resulting in a minimum protein length of 98 amino acids. These ORF finding parameters resulted in a total of 130 461 ORFs across 46 755 transcripts. PSAURON was run with default settings on these 130 461 ORFs. Figure 6 shows the in-frame PSAURON score distribution for these lncRNA ORFs, alongside scores of human CDSs (RefSeq GRCh38.p14) for comparison.

GeneMarkS-T version 5.1 was run with default settings directly on the lncRNA transcripts to establish a baseline performance. GeneMarkS-T reported a total of 32 884 false positive proteins, a false positive rate of 30.7%. To match the behavior of GeneMarkS-T, only the single best scoring ORF was kept for each transcript when using PSAURON. Using PSAURON to score lncRNA ORFs yielded 12 601 false positive proteins, a false positive rate of 11.7%. PSAURON reported 20 283 fewer false positive proteins than GeneMarkS-T.

**Figure 7.**
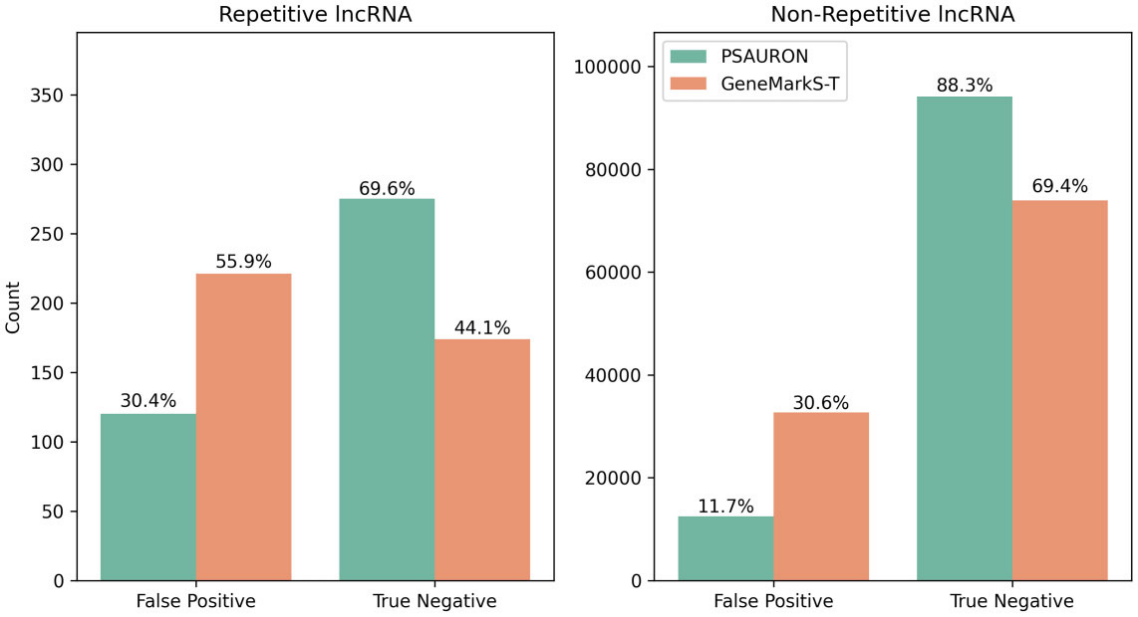
Effect of highly repetitive sequence on lncRNA ORF classification. Transcripts comprised of 50% or more repetitive sequence are shown on the left, while all other lncRNA are shown on the right. Only the top 0.34% most repetitive transcripts were classified as repetitive. Repeats increase the false positive rate of both PSAURON (11.7–30.4%) and GeneMarkS-T (30.6–55.9%).

To test the effect of repetitive sequences on protein prediction, we ran DustMasker version 1.0.0 (23) on the LNCipedia high-confidence lncRNA transcripts. We classified transcripts as repetitive if 50% or more of the sequence was masked by DustMasker. In total, 395 (0.34%) of the 107 039 transcripts were classified as repetitive. As shown in Figure 7, the false positive rate of PSAURON increases from 11.7% to 30.4% in these repetitive transcripts, while GeneMarkS-T finds more false positives than true negatives in repetitive transcripts.

## Discussion

PSAURON is a computational tool that can recognize protein-coding genes in a wide range of eukaryotic species. Similar to Balrog (9), and in contrast to most previous *ab initio* gene finders, PSAURON recognizes features of proteins rather than DNA sequences. Thus, unlike most gene finders, PSAURON does not need to be trained specifically on each species, but only needs to be trained once, as we have done here using a diverse dataset derived from over 1000 plant and animal genomes. PSAURON can evaluate protein sequences and provide confidence scores with no need for organism-specific retraining.

PSAURON can be used to score all proteins in a genome individually and to produce a single overall score. This represents a novel approach for quality assessment of genome annotation in eukaryotes, one that does not require the prior identification of a set of single-copy genes or other species-specific input. This approach addresses a critical need in the field of genomics, as the precision of protein-coding gene annotations is crucial for downstream analyses and applications. As demonstrated by the thousands of low-quality proteins present in the reference rice proteome, problems with annotation workflows may go unnoticed even in the most important model species.

PSAURON can also generate model-based confidence scores for individual proteins, requiring no additional experiments such as proteomics or large-scale manual curation. Curated confidence rankings, such as those available for *Arabidopsis*, are not feasible to produce for every genome sequenced today. The relationship between PSAURON scores and the TAIR10 gene confidence rankings for *Arabidopsis* suggests that PSAURON scores reflect protein annotation quality. By providing a confidence score for each annotated gene, PSAURON can enable researchers to quickly identify potential inaccuracies and prioritize sequences that require either further validation or removal from annotation. We hope PSAURON will provide additional useful information to researchers wishing to produce accurate annotations for a wide range of species.

One unexpected result of our evaluations was that the annotation of rice, arguably humanity’s most important food source (24), received a substantially lower proteome-wide PSAURON score compared to other reference proteomes. This suggests that the current reference rice proteome contains thousands of inaccurate protein annotations. This finding was further supported by the observation that low-PSAURON-scoring rice proteins tend to have low-confidence AlphaFold2 structural predictions, as represented by the abnormal pLDDT distribution of the rice proteome. We have previously shown that pLDDT can be used as a metric to help improve protein annotations, even in highly-studied genomes (19). It is important to note that low pLDDT scores alone are not definitive proof that sequences do not represent real proteins, particularly for those proteins that are inherently unstructured. Still, given the substantially skewed pLDDT distribution of low-PSAURON-scoring proteins in the rice proteome, our results suggest that the rice annotation contains an unusually high number of errors.

Technical and biological noise remains a problem in genome annotation (25). MANE is a high-quality human annotation that has been proposed as the ‘universal standard’ for clinical variant reporting (26). As expected, MANE received one of the highest scores among all annotations tested here, with 97.6% of included proteins receiving a positive PSAURON assessment. Even so, some MANE proteins may need correction (19). Only 83% of 234 ‘uncharacterized’ proteins (i.e. proteins with no known function) in MANE v1.3 scored well according to PSAURON. Additionally, two out of three ‘umcharacterized [*sic*]’ proteins in MANE v1.3 scored poorly according to PSAURON, suggesting that even the most highly curated human gene set is not yet perfect.

In some cases, users may wish to include more proteins in an annotation at the cost of including technical and biological noise (25). The GENCODE human annotation (27), for example, aims to be a comprehensive view of human biology, while MANE restricts itself to a single high-confidence transcript per protein-coding locus (26). Accordingly, MANE receives a higher PSAURON score than GENCODE. A higher score does not necessarily mean that one database is superior. Differences in PSAURON scores may be the result of necessary decisions based on different use cases for each database.

Similarly, predictions of relatively conservative gene models such as AUGUSTUS trained on ‘a highly specific set of high confidence genes’ (28) may produce higher PSAURON scores than a more complex model. A high PSAURON score does not guarantee annotation correctness overall. Any gene annotation pipeline may trade precision for sensitivity, potentially at the cost of overall annotation quality. For example, a higher PSAURON score might be achieved by a short protein with an incorrect downstream start site rather than the full, correctly annotated, protein. Attempts to improve start site annotation are unlikely to benefit from PSAURON.

PSAURON should not be used as the sole oracle of annotation quality. No tool currently exists that can fully evaluate all aspects of genome annotation. Rather, PSAURON is intended to help control annotation noise and highlight potential inaccuracies in annotations, facilitating the identification of sequences that may require further investigation.

In its current form, PSAURON is not intended to be used as an *ab initio* gene finder, because it does not consider all possible combinations of exons and introns in a genomic DNA sequence as some *ab initio* gene finders do (28–31). Because it scores a user-provided annotation, PSAURON cannot estimate the completeness of that annotation. Rather, it can be used as a quality-control measure, serving as an estimate of annotation noise and a means to highlight potentially inaccurate annotations. However, PSAURON could be used as a module in a more general *ab initio* gene finder in future work. Additionally, because PSAURON scores at the amino acid level, we would expect any protein-like sequence to be assigned a high score. This means a pseudogene that retains the correct reading frame when annotated as a protein-coding gene would likely also receive a high score. Thus, other evidence may be required to remove pseudogenes during genome annotation.

Our experiments demonstrated the effectiveness of PSAURON through a series of analyses on reference datasets. As expected, the proteome-wide PSAURON scores for a variety of highly-studied model organisms were high, commensurate with the expected quality of their annotations. Perhaps surprisingly, PSAURON also appeared to be effective for scoring annotation in the bacterium *E. coli* and the archaeon *M. jannaschii*, although its training data did not include organisms from either kingdom. In contrast to eukaryotic genomes, prokaryotic genomes are relatively simple to annotate, particularly when they have low GC content (9). These high scores suggest that PSAURON, which was trained exclusively on eukaryotic protein sequence, learned to recognize more universal features of protein sequences, features that apply to all living species.

In conclusion, PSAURON represents a new method for assessing protein-coding gene annotation across a wide range of eukaryotic species. Its ability to leverage a large, diverse training dataset to assess the quality of protein-coding gene annotations has the potential to improve the reliability of protein-coding gene annotation.

## Supplementary Material

lqae189_Supplemental_Files

## Data Availability

PSAURON is freely available at https://github.com/salzberg-lab/PSAURON and doi.org/10.5281/zenodo.14532182.

## References

[1] Hotaling S., Kelley J.L., Frandsen P.B. Toward a genome sequence for every animal: where are we now?. Proc. Natl Acad. Sci. U.S.A. 2021; 118:e2109019118. 34862323 10.1073/pnas.2109019118 PMC8719868

[2] O’Leary N.A., Wright M.W., Brister J.R., Ciufo S., Haddad D., McVeigh R., Rajput B., Robbertse B., Smith-White B., Ako-Adjei D. et al. Reference sequence (RefSeq) database at NCBI: current status, taxonomic expansion, and functional annotation. Nucleic Acids Res. 2016; 44:D733–D745. 26553804 10.1093/nar/gkv1189 PMC4702849

[3] Rhie A., Walenz B.P., Koren S., Phillippy A.M. Merqury: reference-free quality, completeness, and phasing assessment for genome assemblies. Genome Biol. 2020; 21:245. 32928274 10.1186/s13059-020-02134-9 PMC7488777

[4] Gurevich A., Saveliev V., Vyahhi N., Tesler G. QUAST: quality assessment tool for genome assemblies. Bioinformatics. 2013; 29:1072–1075. 23422339 10.1093/bioinformatics/btt086 PMC3624806

[5] Salzberg S.L., Phillippy A.M., Zimin A., Puiu D., Magoc T., Koren S., Treangen T.J., Schatz M.C., Delcher A.L., Roberts M. et al. GAGE: a critical evaluation of genome assemblies and assembly algorithms. Genome Res. 2012; 22:557–567. 22147368 10.1101/gr.131383.111 PMC3290791

[6] Simão F.A., Waterhouse R.M., Ioannidis P., Kriventseva E.V., Zdobnov E.M. BUSCO: assessing genome assembly and annotation completeness with single-copy orthologs. Bioinformatics. 2015; 31:3210–3212. 26059717 10.1093/bioinformatics/btv351

[7] Nevers Y., Warwick Vesztrocy A., Rossier V., Train C.-M., Altenhoff A., Dessimoz C., Glover N.M. Quality assessment of gene repertoire annotations with OMArk. Nat. Biotechnol. 2024; 10.1038/s41587-024-02147-w. PMC11738984 38383603

[8] Tolkien J.R.R. The Fellowship of the Ring. 1954; United Kingdom George Allen & Unwin.

[9] Sommer M.J., Salzberg S.L. Balrog: a universal protein model for prokaryotic gene prediction. PLoS Comput. Biol. 2021; 17:e1008727. 33635857 10.1371/journal.pcbi.1008727 PMC7946324

[10] Bai S., Zico Kolter J., Koltun V. An empirical evaluation of generic convolutional and recurrent networks for sequence modeling. 2018; arXiv doi:19 April 2018, preprint: not peer reviewed https://arxiv.org/abs/1803.01271.

[11] Akiba T., Sano S., Yanase T., Ohta T., Koyama M. Optuna: a next-generation hyperparameter optimization framework. Proceedings of the 25th ACM SIGKDD International Conference on Knowledge Discovery & Data Mining, KDD ’19. 2019; New York, NY Association for Computing Machinery 2623–2631.

[12] Loshchilov I., Hutter F. Decoupled weight decay regularization. 2017; arXiv doi:14 November 2017, preprint: not peer reviewed https://arxiv.org/abs/1711.05101.

[13] Souvorov A., Kapustin Y., Kiryutin B., Chetvernin V., Tatusova T., Lipman D. Gnomon—NCBI eukaryotic gene prediction tool. 2010; (23 October 2024, date last accessed) https://www.ncbi.nlm.nih.gov/refseq/annotation_euk/gnomon/.

[14] Sakai H., Lee S.S., Tanaka T., Numa H., Kim J., Kawahara Y., Wakimoto H., Yang C.-C., Iwamoto M., Abe T. et al. Rice Annotation Project Database (RAP-DB): an integrative and interactive database for rice genomics. Plant Cell Physiol. 2013; 54:e6. 23299411 10.1093/pcp/pcs183 PMC3583025

[15] Kawahara Y., de la Bastide M., Hamilton J.P., Kanamori H., McCombie W.R., Ouyang S., Schwartz D.C., Tanaka T., Wu J., Zhou S. et al. Improvement of the *Oryza sativa* Nipponbare reference genome using next generation sequence and optical map data. Rice. 2013; 6:4. 24280374 10.1186/1939-8433-6-4 PMC5395016

[16] Jumper J., Evans R., Pritzel A., Green T., Figurnov M., Ronneberger O., Tunyasuvunakool K., Bates R., Žídek A., Potapenko A. et al. Highly accurate protein structure prediction with AlphaFold. Nature. 2021; 596:583–589. 34265844 10.1038/s41586-021-03819-2 PMC8371605

[17] Varadi M., Anyango S., Deshpande M., Nair S., Natassia C., Yordanova G., Yuan D., Stroe O., Wood G., Laydon A. et al. AlphaFold Protein Structure Database: massively expanding the structural coverage of protein-sequence space with high-accuracy models. Nucleic Acids Res. 2022; 50:D439–D444. 34791371 10.1093/nar/gkab1061 PMC8728224

[18] Tunyasuvunakool K., Adler J., Wu Z., Green T., Zielinski M., Žídek A., Bridgland A., Cowie A., Meyer C., Laydon A. et al. Highly accurate protein structure prediction for the human proteome. Nature. 2021; 596:590–596. 34293799 10.1038/s41586-021-03828-1 PMC8387240

[19] Sommer M.J., Cha S., Varabyou A., Rincon N., Park S., Minkin I., Pertea M., Steinegger M., Salzberg S.L. Structure-guided isoform identification for the human transcriptome. eLife. 2022; 11:e82556. 36519529 10.7554/eLife.82556 PMC9812405

[20] Lamesch P., Berardini T.Z., Li D., Swarbreck D., Wilks C., Sasidharan R., Muller R., Dreher K., Alexander D.L., Garcia-Hernandez M. et al. The *Arabidopsis* Information Resource (TAIR): improved gene annotation and new tools. Nucleic Acids Res. 2012; 40:D1202–D1210. 22140109 10.1093/nar/gkr1090 PMC3245047

[21] Volders P.-J., Helsens K., Wang X., Menten B., Martens L., Gevaert K., Vandesompele J., Mestdagh P. LNCipedia: a database for annotated human lncRNA transcript sequences and structures. Nucleic Acids Res. 2013; 41:D246–D251. 23042674 10.1093/nar/gks915 PMC3531107

[22] Tang S., Lomsadze A., Borodovsky M. Identification of protein coding regions in RNA transcripts. Nucleic Acids Res. 2015; 43:e78. 25870408 10.1093/nar/gkv227 PMC4499116

[23] Morgulis A., Gertz E.M., Schäffer A.A., Agarwala R. A fast and symmetric DUST implementation to mask low-complexity DNA sequences. J. Comput. Biol. 2006; 13:1028–1040. 16796549 10.1089/cmb.2006.13.1028

[24] Childs N. Rice sector at a glance. 2023; (23 October 2024, date last accessed) https://www.ers.usda.gov/topics/crops/rice/rice-sector-at-a-glance/.

[25] Eling N., Morgan M.D., Marioni J.C. Challenges in measuring and understanding biological noise. Nat. Rev. Genet. 2019; 20:536–548. 31114032 10.1038/s41576-019-0130-6 PMC7611518

[26] Morales J., Pujar S., Loveland J.E., Astashyn A., Bennett R., Berry A., Cox E., Davidson C., Ermolaeva O., Farrell C.M. et al. A joint NCBI and EMBL-EBI transcript set for clinical genomics and research. Nature. 2022; 604:310–315. 35388217 10.1038/s41586-022-04558-8 PMC9007741

[27] Frankish A., Carbonell-Sala S., Diekhans M., Jungreis I., Loveland J.E., Mudge J.M., Sisu C., Wright J.C., Arnan C., Barnes I. et al. GENCODE: reference annotation for the human and mouse genomes in 2023. Nucleic Acids Res. 2023; 51:D942–D949. 36420896 10.1093/nar/gkac1071 PMC9825462

[28] Gabriel L., Brůna T., Hoff K.J., Ebel M., Lomsadze A., Borodovsky M., Stanke M. BRAKER3: fully automated genome annotation using RNA-seq and protein evidence with GeneMark-ETP, AUGUSTUS and TSEBRA. Genome Res. 2024; 34:769–777. 38866550 10.1101/gr.278090.123 PMC11216308

[29] Brůna T., Hoff K.J., Lomsadze A., Stanke M., Borodovsky M. BRAKER2: automatic eukaryotic genome annotation with GeneMark-EP+ and AUGUSTUS supported by a protein database. NAR Genom. Bioinform. 2021; 3:lqaa108. 33575650 10.1093/nargab/lqaa108 PMC7787252

[30] Banerjee S., Bhandary P., Woodhouse M., Sen T.Z., Wise R.P., Andorf C.M. FINDER: an automated software package to annotate eukaryotic genes from RNA-seq data and associated protein sequences. BMC Bioinformatics. 2021; 22:205. 33879057 10.1186/s12859-021-04120-9 PMC8056616

[31] Holt C., Yandell M. MAKER2: an annotation pipeline and genome-database management tool for second-generation genome projects. BMC Bioinformatics. 2011; 12:491. 22192575 10.1186/1471-2105-12-491 PMC3280279

