# Supplementary material for: PSAURON: a tool for assessing protein annotation across a broad range of species
Source: NAR Genom Bioinform. 2025 Jan 7;7(1):lqae189. doi: 10.1093/nargab/lqae189 (PMC11704789; doi:10.1093/nargab/lqae189)
Supplement: lqae189_Supplemental_Files [file lqae189_supplemental_files.zip › Supplementary Legends.docx]

Supplemental Table 1: NCBI accession numbers of plant and animal genomes used to train the PSAURON TCN model.

Supplemental Table 2: PSAURON output for proteins in MANE v1.3.

Supplemental Table 3: PSAURON output for proteins in the UniProt reference rice proteome UP000059680.
